# An Investigation into the Friction Stir Spot Welding Behavior of 3D-Printed Glass Fiber-Reinforced Polylactic Acid

**DOI:** 10.3390/polym18091041

**Published:** 2026-04-24

**Authors:** Emre Kanlı, Oğuz Koçar, Nergizhan Anaç

**Affiliations:** Department of Mechanical Engineering, Faculty of Engineering, Zonguldak Bülent Ecevit University, Zonguldak 67100, Türkiye; emreknli@hotmail.com (E.K.); nergizhan.kavak@beun.edu.tr (N.A.)

**Keywords:** 3D printing, glass fiber-reinforced polylactic acid, friction stir spot welding, mechanical properties

## Abstract

The production of fiber-reinforced polymer composites using 3D printing technology offers significant potential and opportunities for industrial applications. However, current dimensional limitations in 3D printing necessitate the use of joining techniques to obtain larger components. Recently, innovative strategies such as friction stir spot welding (FSSW) have attracted considerable attention for joining polymer composites due to their ability to produce strong joints with relatively low heat input (solid-state welding). Nevertheless, it is important to understand how the fibers present in fiber-reinforced polymer composites influence material flow and welding performance during the FSSW process. In this study, glass fiber-reinforced polylactic acid (PLA-GF) composite samples produced using a 3D printer were joined by means of FSSW. Five different tool rotational speeds (900, 1200, 1500, 1800, and 2100 rpm) and three different plunge rates (10, 20, and 30 mm/min) were employed during the welding process. Mechanical tests were performed on the welded joints to investigate the relationship between the welding parameters and the resulting mechanical properties. In addition, microstructural analyses were conducted to examine the formation of welding defects. The results revealed that three distinct zones were formed in the material after the FSSW process: the stir zone, mixed zone, and shoulder zone. Defects were observed in the mixed zone of the samples exhibiting relatively lower mechanical properties. The highest tensile force was achieved at a plunge rate of 20 mm/min and a rotational speed of 900 rpm. The highest bending force, on the other hand, was obtained at a plunge rate of 30 mm/min and a tool rotational speed of 2100 rpm.

## 1. Introduction

3D printing has become a transformative technology in manufacturing by enabling the production of components with complex geometries that are difficult or impossible to fabricate using conventional methods [1]. With 3D printers, it is possible to produce interconnected parts in a single manufacturing step without the need for additional components [2]. However, the dimensions of the manufactured parts are limited by the build platform size of the printer. Consequently, the joining of such parts is generally performed using conventional techniques such as adhesive bonding and welding, or hybrid joining methods derived from these approaches [3]. Among these techniques, welding enables the formation of stronger and more durable joints that exhibit greater resistance to adverse environmental conditions between the joined materials [4,5]. For this reason, research on the weldability of thermoplastic parts produced by 3D printing has attracted increasing attention.

Friction stir spot welding is a hybrid welding technique developed based on the principles of Resistance Spot Welding and Friction Stir Welding (FSW). FSSW is a solid-state welding process in which overlapped parts are joined at a single point. The method has been used in manufacturing since the early 1990s. Similar to FSW, the tool used in the FSSW process consists of a shoulder and a stirring pin. The heat required for welding is generated by the friction between the tool shoulder and the pin with the workpiece as the tool plunges into the material. As a result of friction and stirring, the overlapped materials soften and mix with each other. Compared with conventional fusion welding methods, the FSSW process minimizes thermal distortion and preserves the chemical, mechanical, and thermal properties of the materials [6].

Iftikhar et al. [7] aimed to understand the potential of friction stir-based welding techniques. In their study, they investigated the mechanical, thermal, and chemical behavior of recycled High-Density Polyethylene (HDPE) after the FSSW process. Their results indicated that recycled thermoplastics can be used to produce sustainable lightweight structures through FSW/FSSW methods and may have potential applications in industry. In the existing literature, there are several studies in which FSSW has been used to join thermoplastic materials [8,9,10,11,12,13,14]. However, studies focusing on the FSSW of materials produced by 3D printing technology remain limited. In 3D printers, Polylactic Acid (PLA) and Acrylonitrile Butadiene Styrene (ABS) are commonly used materials. Nevertheless, filament manufacturers are continuously expanding their product range. With the development of new printing materials, manufacturers offer alternative solutions for engineering applications. In the future, it is expected that reinforced composites and high-performance materials compatible with 3D printing will play a greater role in industrial applications [15,16]. Therefore, evaluating the FSSW performance of 3D-printed thermoplastic materials is considered important.

Baysal et al. [17] investigated the FSSW of PLA-based materials produced by 3D printing, specifically PLA Plus and carbon fiber-reinforced PLA (PLA-CF). In their study, both single-material (PLA Plus/PLA Plus, PLA-CF/PLA-CF) and multi-material (50% PLA Plus/50% PLA-CF) specimens were examined. The results indicated that the mechanical performance of the joints improved when appropriate material positioning was ensured, and the welding parameters were optimized. Furthermore, the findings revealed that multi-material joints exhibited better performance than single-material joints. In another study [18], the FSSW technique was employed to join additively manufactured chopped carbon fiber-reinforced nylon-based composites. As a result of the FSSW process, the joints exhibited good mechanical performance. Similar improvements were also reported by Tiwary et al. [19] in their investigation of FSSW applied to ABS/PLA 3D-printed components. Singh et al. [20] examined the use of joining techniques in the repair of 3D-printed biomedical scaffolds. In their study, PLA reinforced with poly ether ketone ketone (PEKK), hydroxyapatite (HAp), and chitosan (CS) was produced using Fused Deposition Modeling (FDM) and subsequently joined using the FSSW technique.

From a general perspective, the performance of fiber-reinforced polylactic acid (PLA)-based materials manufactured by conventional techniques differs from that of materials produced by 3D printing/additive manufacturing. The fibers added to the matrix exhibit distinctive behavior during melting and flow processes due to fiber orientation and interfacial adhesion/debonding. The most widely used reinforcement materials in industry are carbon fiber (CF) and glass fiber (GF). However, the main difference arising from the incorporation of these reinforcements into PLA is related to thermal conductivity and heat dissipation.

The low thermal conductivity of glass fiber (GF) may hinder the dissipation of friction-generated heat into the material. In friction stir spot welding (FSSW), this may lead to overheating of the PLA matrix at the glass fiber/matrix interface, thereby causing a reduction in mechanical properties.

In PLA-CF materials, the heat dissipation capability of carbon fibers may provide an advantage, whereas in PLA-GF materials, the insulating nature of glass fibers may create a risk of localized thermal accumulation in the weld zone [21,22,23,24,25,26].

This study investigated the influence of the FSSW process on the mechanical behavior of 3D-printed glass fiber-reinforced polylactic acid parts. Among the FSSW process parameters, the plunge rate (10, 20, and 30 mm/min) and tool rotational speed (900, 1200, 1500, 1800, and 2100 rpm) were varied, while the pin geometry was kept constant as square. The relationship between the tensile-shear and bending force obtained after welding and the microstructural features of the weld zone, together with the characteristics of the resulting joint, was analyzed. This study aims to provide guidance for optimizing FSSW parameters in the joining of fiber-reinforced thermoplastic materials manufactured by 3D printing, and to enhance understanding of the applicability of the FSSW technique for joining 3D-printed parts.

## 2. Materials and Methods

### 2.1. Filament Properties

PLA is a widely used material in 3D printing due to its low melting temperature, ease of printability, low toxicity, and cost advantages. However, it has relatively low impact toughness and softens at approximately 60–65 °C [27,28]. These limitations have led to the need to improve the properties of PLA by incorporating reinforcement materials, particularly for structural and engineering applications [28,29]. Through the addition of various fillers and reinforcements, improvements in impact strength and ductility, flow behavior and interlayer adhesion, as well as surface quality and dimensional stability are aimed [30]. For this reason, several PLA-based filament variants such as PLA-CF (carbon fiber-reinforced), PLA-Wood (wood-filled), and PLA-GF are commercially available [31,32]. PLA-GF benefits from the ease of printability of PLA while also providing enhanced thermal resistance and good wear resistance due to the presence of glass fiber reinforcement, making it suitable for applications where improved thermal and mechanical performance is required [29,33,34]. PLA GF materials can be used for rigid, heat-resistant functional prototypes and end-use parts. Common applications include industrial tools, jigs, and fixtures, as well as automotive interior parts [34].

White glass fiber reinforced PLA filament with a diameter of 1.75 mm, manufactured by eSUN, was used as the printing material. The mechanical properties of the composite filament, produced by reinforcing a standard PLA matrix with 16% glass fiber, are presented in Table 1.

### 2.2. Printing Parameters

As part of the experimental study, friction stir spot welding (FSSW) plates were fabricated from PLA-GF filament. The specimens were produced using a Bambu Lab X1E model 3D printer (Bambu Lab, Shenzhen, China) based on the Fused Deposition Modeling (FDM) process. Its hardened steel nozzle system helps minimize printing defects and reduces nozzle wear, particularly during the processing of abrasive reinforced materials such as glass fiber-filled filaments [36,37]. All samples were printed horizontally on the build plate without support structures, using a 0.4 mm diameter nozzle.

Prior to the 3D printing process, the slicing operation was performed using the BambuStudio v2.0.3 slicing software released by the 3D printer manufacturer. The parts were produced with the following parameters: 0.2 mm layer thickness, 100% infill ratio, 80 mm/s print speed, 55 °C bed temperature, and 220 °C nozzle temperature. The specimens were printed using a linear infill pattern with a raster angle of [−45, +45] and a wall count of three. The production parameters are given in Table 2.

### 2.3. Determination of FSSW Parameters

In FSSW, weld quality is affected by many parameters. The most effective parameters in FSSW are stated as tool rotational speed, plunge rate, dwell time, tool geometry, shoulder diameter and shoulder profile (flat, concave, grooved, etc.), pin diameter, pin length and pin profile (cylindrical, conical, triangular, screw, etc.) and tool material [13,38,39,40,41]. Table 3 discusses the FSSW parameters and their effects on weld quality.

Table 4 presents the FSSW parameters used in the literature for FSSW. Examination of the studies reveals that FSSW parameters vary depending on the material type. This also indicates that there is no single method for determining welding parameters and that experimental studies are needed to identify suitable parameters for improving welding quality.

In line with previous studies [17] and general evaluations, the FSSW parameters used in the experiments for PLA-GF are given in Table 5. During the FSSW process, the tool rotational speed and tool plunge rate were varied, while the stirring time of 10 s, dwell time of 10 s, shoulder plunge depth of 0.2 mm, and tool plunge depth of 4.6 mm were kept constant.

### 2.4. Experimental Setup

**Figure 1:**
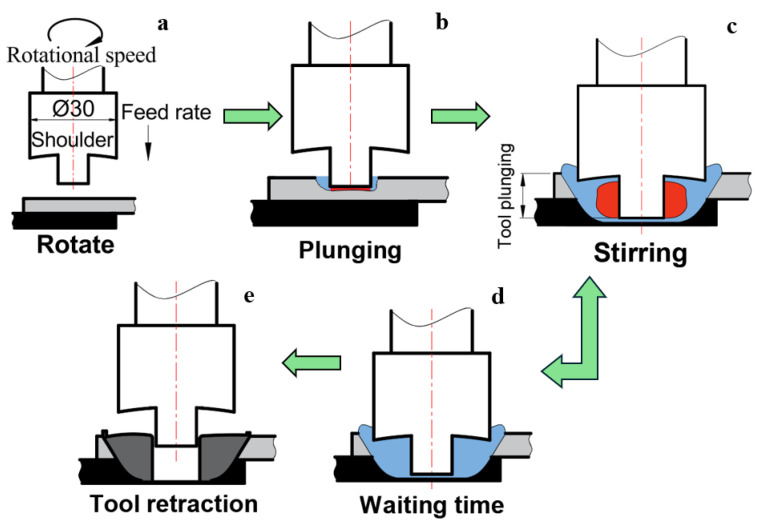
Schematic representation of the FSSW process parameters and operational stages: (**a**) First step: Initiation of tool rotation and downward movement; (**b**) Second step: Plunging of the tool into the workpiece; (**c**) Third step: Stirring phase; (**d**) Fourth step: Dwelling at the final position for a specified duration; and (**e**) Fifth step: Withdrawal of the tool.

For the FSSW process, a Spinner U630 CNC milling machine (Spinner, Sauerlach, Germany, 22 kW, 12,000 rpm, Y-axis 530 mm, Z-axis 465 mm, and a table size of 650 mm) and a BT40 tool holder were used. To ensure that the plates remained fixed during the FSSW process, a fixture connection compatible with the plate dimensions was mounted onto the CNC table. After each operation, the stirring pin was checked, and the tool was allowed to cool before the next experiment. The FSSW process steps are presented in Figure 1. In Figure 1a, the tool movements are illustrated; the remainder of the figure features the following: in (b), the contact between the tool and the workpiece; in (c), stationary stirring at the plunge depth for 10 s; in (d), the dwell period with the tool motion halted (10 s); and finally, in (e), the withdrawal of the tool is schematically represented.

**Figure 2:**
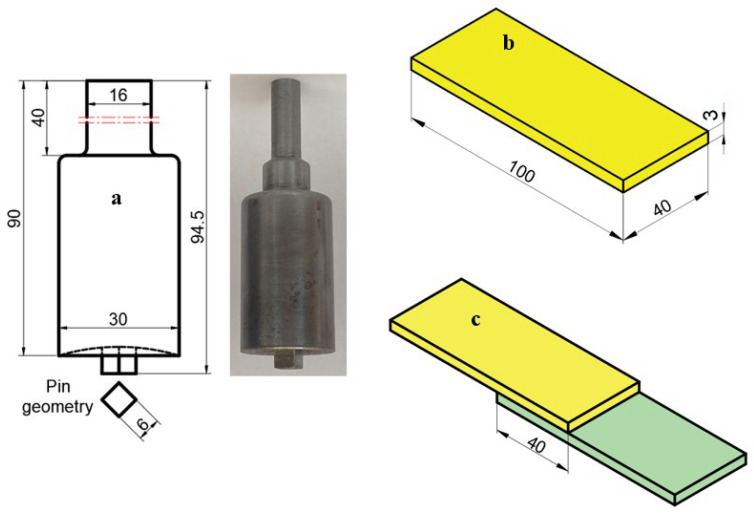
View of the mixer tip, FSSW sample, and lap joint: (**a**) Tool dimensions, (**b**) specimen dimensions for FSSW, and (**c**) lap joint configuration (all dimensions are in mm).

Figure 2 presents the stirring tool geometry and tool dimensions (a), along with the FSSW specimen dimensions (b) and the lap joint configuration dimensions (c). The tool shoulder diameter was 30 mm with a shoulder height of 40 mm, while the shoulder plunge depth was set to 0.2 mm. The plunge depth of the stirring pin was 4.7 mm, and a square pin geometry was preferred. To prevent the expulsion of the plasticized material during welding and to ensure enhanced material consolidation within the weld zone, the tool shoulder was designed with a concave profile. The stirring pin was fabricated from 2379 tool steel, a ledeburitic cold work tool steel containing approximately 12% chromium.

### 2.5. Tests Used in the Study

**Figure 3:**
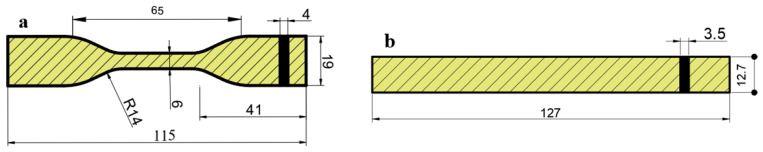
Technical drawings of the tensile (**a**) and bending (**b**) test specimens (dimensions in mm).

To determine the mechanical strength of the reference and welded specimens, tensile tests were conducted in accordance with ASTM D638 [54], and three-point bending tests were performed following ASTM D790 [55]. The dimensions of the tensile and bending test specimens are presented in Figure 3. Tensile-shear tests were carried out at a crosshead speed of 1 mm/min, while bending tests were conducted at 5 mm/min, both at room temperature and with four repetitions. In addition, images were obtained from the weld zone, and microstructural changes depending on the processing parameters were analyzed.

Microstructural observations of both reference and welded specimens were performed using a scanning electron microscope (SEM), specifically a FEI Quanta FEG 250 model (Hillsboro, OR, USA). Prior to SEM analysis, all specimens were coated with a gold–palladium layer to enhance conductivity.

## 3. Conclusions and Discussion

**Figure 4:**
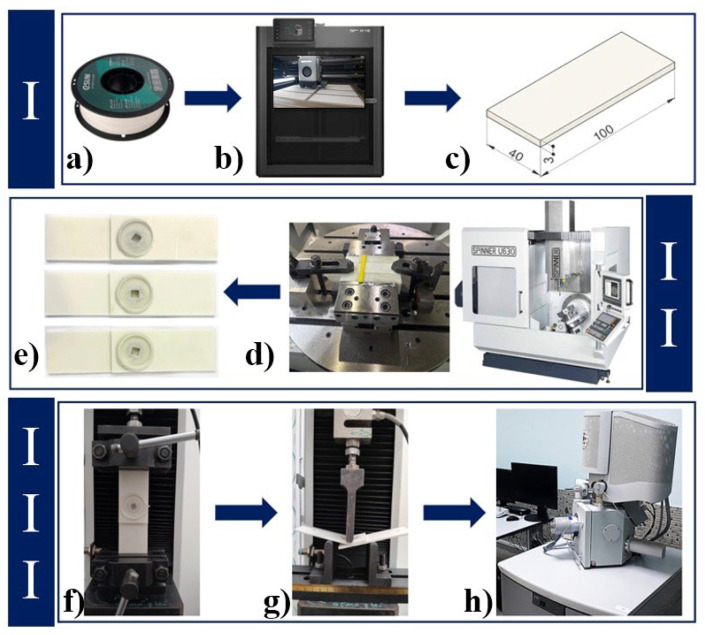
Flow chart of the printing process, FSSW procedure, and testing stages (**a**) Filament, (**b**) 3D printing process, (**c**) FSSW sample dimensions, (**d**) FSSW process, (**e**) Image of the weld joint after FSSW, (**f**) Tensile test, (**g**) Bending test, (**h**) Microstructure imaging process.

This study consists of three main stages: fabrication of the specimens, application of the FSSW process, and evaluation of weld quality. Plates were produced using PLA-GF filament with a 100% infill ratio and a layer thickness of 0.2 mm (Figure 4I). For the joining of the plates, the FSSW process parameters included five different tool rotational speeds (900, 1200, 1500, 1800, and 2100 rpm), three different plunge rates (10, 20, and 30 mm/min), a stirring time of 10 s, a shoulder plunge depth of 0.2 mm, and a tool plunge depth of 4.6 mm (Figure 4II). The joint efficiency of PLA-GF plates produced via the FSSW method was determined, and the effects of plunge rate and tool rotational speed on weld quality were investigated. Microstructural images of the weld zone were obtained, and the results derived from the varying parameters were compared with those reported in the existing literature (Figure 4III).

In the FSSW process, heat input is primarily governed by the tool rotational speed and the plunge rate. An increase in rotational speed enhances the friction between the tool and the material, leading to higher heat generation and improved plasticization of the PLA matrix. However, excessive heat input may result in over-softening of the material, material expulsion (flash), and a reduction in weld quality.

The plunge rate controls the contact time between the tool and the material. At lower plunge rates, the extended contact duration leads to increased heat generation, whereas at higher plunge rates, the reduced contact time results in lower heat input. Therefore, an optimal balance between rotational speed and plunge rate is essential to achieve a sound weld.

In glass fiber-reinforced PLA, the presence of glass fibers restricts the mobility of polymer chains during the stirring process. Compared to neat PLA, this may hinder the plasticization of PLA-GF at the same temperature. Furthermore, glass fibers may obstruct material flow during stirring, making it more difficult to achieve a homogeneous mixture.

Nevertheless, when appropriate process parameters are selected, the redistribution of fibers within the softened PLA matrix may be achieved. This can enhance mechanical interlocking and improve joint strength. However, non-uniform fiber distribution or fiber agglomeration may lead to void formation and weak bonding regions, ultimately resulting in reduced weld strength [56,57,58,59].

### 3.1. Mechanical Properties of the Base Material

**Figure 5:**
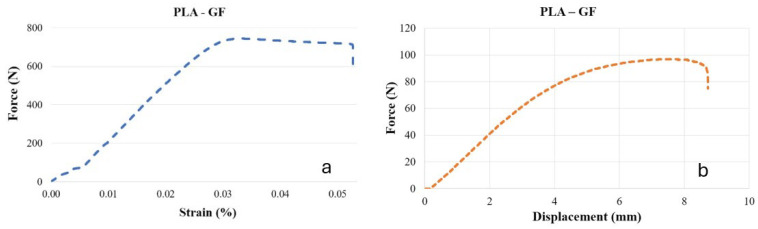
Tensile-shear and three-point bending test curves: (**a**) Force–strain curve, (**b**) Force–displacement curve.

The force–strain curve obtained from the tensile test of PLA-GF is presented in Figure 5a, while the force–displacement curve from the bending test is shown in Figure 5b. For the reference (Ref.) PLA-GF material, the tensile force was determined as 726.8 ± 9.3 N (corresponding to 30.2 MPa), with an elongation of 4.9%. In the bending test, the bending force was measured as 96.7 ± 0.9 N, and the displacement value was 8.7 ± 0.3 mm. Robinson et al. investigated the mechanical properties of PLA reinforced with 16% glass fiber under different infill ratios (50%, 70%, and 90%) and infill patterns (cubic, octet, and cubic subdivision). The results indicated that the cubic pattern exhibited the highest tensile strength (24.65 MPa), whereas the octet structure showed superior flexural stiffness [60].

Similarly, Gök and Akay reinforced PLA and ABS materials with 1%, 3%, and 5% carbon fiber and glass fiber additives and produced various composites using a 3D printer. Among the ABS-based composites, the highest tensile strength was obtained for ABS + 3% CF samples (29.67 MPa), while for PLA-based composites, the highest tensile strength was reported for PLA + 3% GF samples (17.47 MPa) [61]. Valean et al. incorporated different fillers into the PLA matrix (GF: 15%, CF: 10–15%, and bronze particles (BP): 20%). The tensile strengths were reported as 39.72 MPa for pure PLA, 27.16 MPa for PLA-GF, 31.15 MPa for PLA-CF, and 10.81 MPa for PLA-BP [62]. The literature demonstrates that reinforcing the PLA matrix with various fillers is a widely studied approach. Among these, carbon fiber and glass fiber are the most commonly used reinforcements. Since the properties of the fillers directly affect their adhesion to the matrix, they significantly influence the mechanical performance of the composite. Therefore, variations in mechanical properties are commonly observed among glass fiber-reinforced PLA materials reported in the literature.

**Figure 6:**
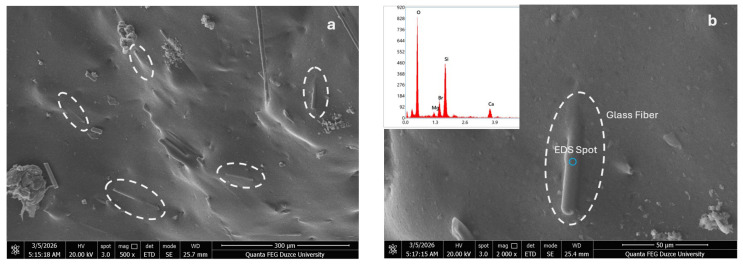
SEM images of PLA/GF composites: (**a**) GF dispersion; (**b**) PLA and GF interface in the 3D-printed sample.

Figure 6 illustrates the condition of the fibers within the PLA matrix. In Figure 6a, the overall distribution of the glass fibers (GF) is presented, while Figure 6b shows the interfacial region between the PLA matrix and GF, along with the results of the energy-dispersive X-ray spectroscopy (EDS) analysis. It is observed that the interfacial bonding between the PLA matrix and the fibers is strong. However, if the filaments are produced or stored under unsuitable conditions, the interfacial adhesion between the matrix and the fibers may be weakened [63,64]. According to the EDS analysis of the glass fibers shown in Figure 6b, the elemental composition was identified as oxygen (70.7 wt%), silicon (20.4 wt%), calcium (4.9 wt%), and bromine (4.0 wt%).

### 3.2. Visual Inspection

**Figure 7:**
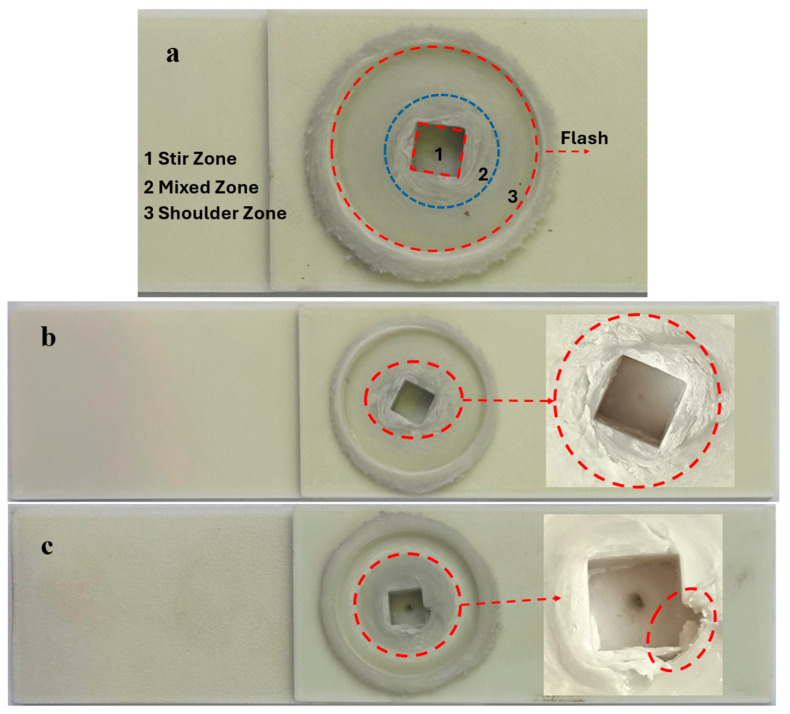
Post-FSSW appearance of selected specimens: (**a**) Identification of the regions formed during FSSW, (**b**) Experiment 6 (weld force: 930.72 N), and (**c**) Experiment 2 (weld force: 731.35 N).

Figure 7a illustrates the regions formed after the FSSW process. The outermost region is the shoulder zone, which is formed due to the contact between the tool shoulder and the material. Since the shoulder penetrates into the workpiece, a slight reduction in thickness occurs. Just outside the shoulder zone, the compressed material accumulates along the outer periphery of the shoulder. The second region, formed by the rotational motion of the tool, is the mixed zone. In this region, the material is plasticized by the heat generated through shoulder contact and tool rotation, and subsequently mixed and re-solidified due to the rotational action of the tool. Material flow patterns aligned with the direction of tool rotation can be observed in the mixed zone.

The final region is the stir zone, where the tool pin actively rotates. After the tool is removed, a cavity remains in this region. While heat generation primarily occurs in the shoulder zone, material flow is more pronounced in areas close to the pin. Under unsuitable FSSW parameters, defects were observed in the mixed zone, which can be attributed to insufficient plasticization and, consequently, inadequate material mixing.

Figure 7b,c present the post-FSSW appearances of the best (Experiment 6) and worst (Experiment 2) specimens, respectively. In Experiment 6 (Figure 7b), the pin geometry in the stir zone is well-formed, and the flow lines generated by mixing in the mixed zone are clearly visible. Additionally, less flash formation is observed compared to Experiment 2. In contrast, Figure 7c shows that defects are present in the stir zone. Furthermore, no distinct mixing patterns are observed in the mixed zone. This can be explained by the use of inappropriate FSSW parameters, which failed to provide the necessary conditions for proper welding.

### 3.3. Results of Tensile Test After FSSW

Table 6 presents the tensile force values as a function of tool rotational speed and plunge rate. The highest tensile force was recorded as 930.7 N at a plunge rate of 20 mm/min and a tool rotational speed of 900 rpm (Experiment 6). At the same rotational speed, the tensile force values were 898.3 N for a plunge rate of 10 mm/min (Experiment 1) and 772.3 N for 30 mm/min (Experiment 11).

The lowest weld force was determined to be 731.4 N at a plunge rate of 10 mm/min and a tool rotational speed of 1200 rpm (Experiment 2). As the plunge rate increased to 20 and 30 mm/min, the tensile force increased to 914.3 N and 907.1 N, respectively. At 1500 rpm, the tensile force values were measured as 832.8 N, 863.2 N, and 837.4 N for plunge rates of 10, 20, and 30 mm/min, respectively. At 1800 rpm, these values were 835.5 N, 864.5 N, and 822.5 N. At 2100 rpm, the highest value was obtained at 20 mm/min with 925.4 N, while the values at the same rotational speed were 795.9 N for 10 mm/min and 869.8 N for 30 mm/min. It can be inferred that, at this tool rotational speed, variations in plunge rate tend to balance their overall effect on tensile force.

**Figure 8:**
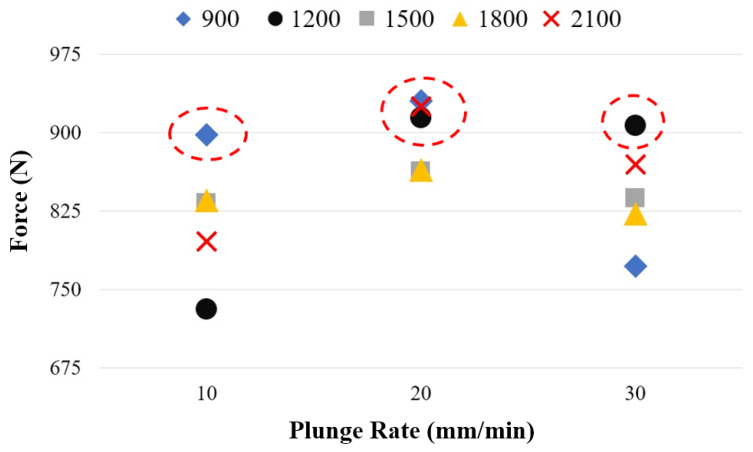
Variation in welding force according to tool rotation speed.

Figure 8 presents the variation in weld force as a function of tool rotational speed when the plunge rate is kept constant. At a plunge rate of 10 mm/min, higher weld forces were obtained at lower tool rotational speeds (900 rpm). Weld quality is directly influenced by the heat generated during the FSSW process. Sufficient heat enables proper plasticization of the material and promotes adequate material flow [39,65,66]. In FSSW, heat generation is directly affected by the interaction between the tool plunge rate and the tool rotational speed. Therefore, optimization of these parameters is critical.

Paoletti et al. investigated the variation in force and torque during the FSSW of polycarbonate plates. They employed process parameters including tool plunge rates of 8 and 34 mm/min, tool rotational speeds of 900 and 2150 rpm, and dwell times of 0 and 21 s. Their results indicated that increasing the plunge rate reduces the interaction time between the tool and the material, thereby decreasing the generated temperature. Conversely, increasing the tool rotational speed enhances the interaction between the tool and material, leading to higher temperatures and improved material softening. However, excessively high rotational speeds may cause excessive plasticization, resulting in material expulsion and thickness reduction [67].

The highest weld forces were obtained at a plunge rate of 20 mm/min for tool rotational speeds of 900 rpm, 1200 rpm, and 2100 rpm, and at a plunge rate of 30 mm/min for a rotational speed of 1200 rpm. Overall, the weld force ranged between 730 and 900 N at 10 mm/min, 860 and 930 N at 20 mm/min, and 770 and 910 N at 30 mm/min. It is evident that at a plunge rate of 20 mm/min, the weld force values are more consistently distributed within a narrower range, indicating that this parameter is more suitable for PLA-GF applications.

**Figure 9:**
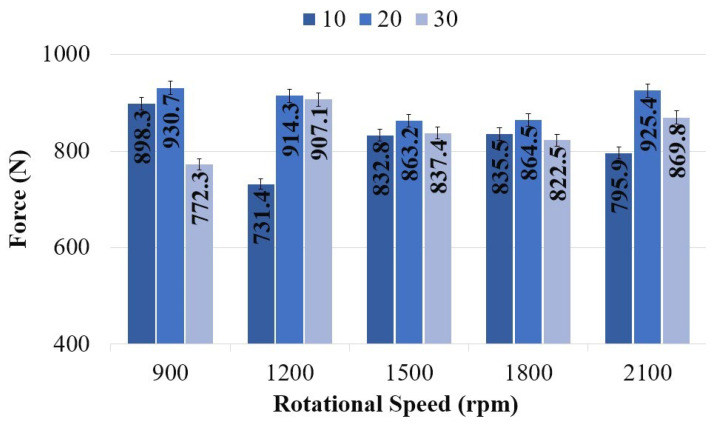
Variation in welding force depending on the feed rate.

Figure 9 presents the variation in weld force as a function of plunge rate at constant tool rotational speeds. At 1500 rpm and 1800 rpm, the weld force values are observed to be relatively similar across all plunge rates, indicating that the effect of plunge rate diminishes at these rotational speeds. The lowest weld forces were determined as 772.3 N at 900 rpm with a plunge rate of 30 mm/min, 731.4 N at 1200 rpm with a plunge rate of 10 mm/min, and 795.9 N at 2100 rpm with a plunge rate of 10 mm/min. These results suggest a clear interaction between tool rotational speed and plunge rate. Accordingly, it can be inferred that at lower rotational speeds, lower plunge rates are more favorable, whereas at higher rotational speeds, higher plunge rates are required to maintain a balance between heat input and material flow. The literature also emphasizes the importance of selecting appropriate process parameters to achieve a balance between heat input and material flow. Ramya et al. investigated the weldability of AA7075 and mild steel using FSSW through two experimental sets. In the first set, the plunge rate was kept constant at 4 mm/min while five different tool rotational speeds (800, 850, 900, 950, and 1000 rpm) were applied. In the second set, the tool rotational speed was fixed at 900 rpm while five different plunge rates (2, 3, 4, 5, and 6 mm/min) were used. The highest weld strength was obtained at 1000 rpm and a plunge rate of 6 mm/min. They concluded that optimization of process parameters is essential to achieve appropriate heat input and effective material flow during the FSSW process [45]. Ahmed et al. investigated the influence of tool rotational speed on the FSSW joining behavior of AA5052-H32 plates. They reported that increasing the tool rotational speed from 500 rpm to 1500 rpm raised the heat input from 1525 J to 4500 J. However, as the rotational speed increased, the weld force decreased (4330 N, 3000 N, and 2569 N, respectively). They concluded that the heat generated at 500 rpm was sufficient for effective welding, whereas excessive heat input led to a reduction in joint strength. As a result, they explained that the increase in tool rotational speed led to higher heat input, which in turn increased material softening [68].

**Figure 10:**
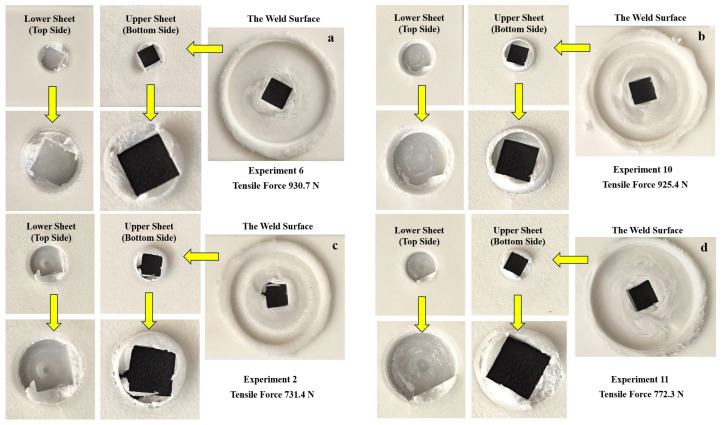
The fracture surfaces of the lap joint test samples: (**a**) Experiment 6; (**b**) Experiment 10; (**c**) Experiment 2; (**d**) Experiment 11.

Figure 10a,b present the fracture surface morphologies of the friction stir spot-welded joints with the highest weld quality (Experiment 6 and Experiment 10), while Figure 10c,d show those with the lowest weld quality (Experiment 2 and Experiment 11). A black background was used in the images to facilitate clearer observation of the damage in the weld region.

In these specimens, the upper and lower sheets were separated after testing. However, with the detachment of the weld zone from the lower sheet, a portion of the upper sheet remained attached to the lower sheet within the nugget region. It is evident that a greater amount of the upper sheet material remained in the nugget region in the specimens shown in Figure 10a,b, compared to those in Figure 10c,d. This can be seen in the spot-like image located in the center of the keyhole on the lower sheet.

This behavior may be attributed to insufficient heat generation and irregular particle distribution within the stir zone in the specimens shown in Figure 10c,d [69]. In contrast, the welding parameters used in Figure 10a,b resulted in improved material mixing within the weld zone. Furthermore, specimens with lower strength exhibited a higher amount of weld flash, which is consistent with findings reported in previous studies [7,70].

**Figure 11:**
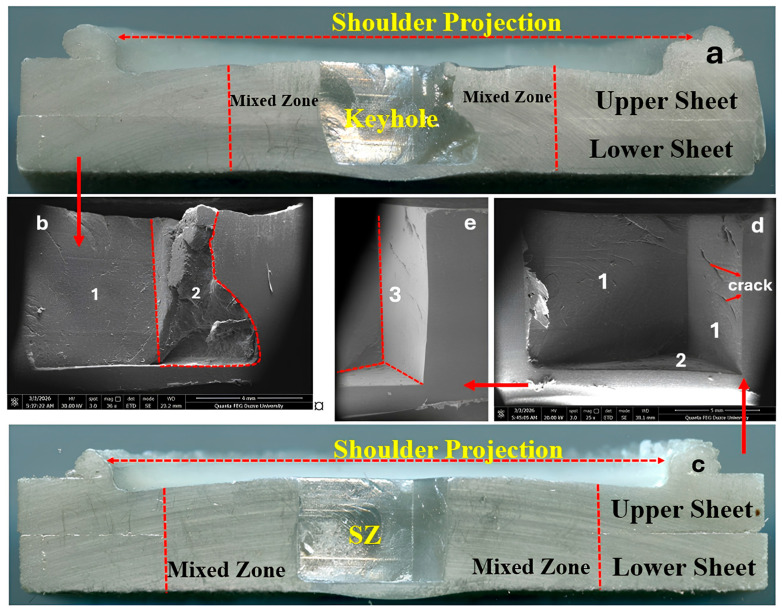
Cross-sectional micrograph of samples: (**a**,**b**) Experiment 2 (10 mm/min and 1200 rpm); (**c**–**e**) Experiment 6 (20 mm/min and 900 rpm).

Figure 11a presents the microstructure of Experiment 2, while Figure 11b shows the corresponding SEM images. In Figure 11a, the mixed zone, keyhole, and shoulder projection are shown. It is observed that the lap interface between the plates disappears in the mixed zone due to the combined effects of temperature and material mixing. A reduction in thickness is evident at the region where the tool shoulder penetrates, and weld flash formation is observed at the edge of the shoulder as a result of the outward flow of the softened material. In Figure 11b, the regions marked as (1) and (2) represent areas where the tool pin directly contacted the material. The microstructural analysis of the specimen joined at a plunge rate of 10 mm/min and a tool rotational speed of 1200 rpm reveals the presence of pits and large voids (Figure 11b, region (2)). This can be attributed to the use of unsuitable FSSW parameters, which led to non-uniform mixing and consequently insufficient material consolidation within the weld zone.

Figure 11c presents the microstructure of Experiment 6, while Figure 11d,e show the corresponding SEM images. Examination of the figures reveals that the stir zone (SZ) boundaries formed in Experiment 6 exhibit a well-defined and regular geometry. In Figure 11d,e, regions labeled (1) and (3) correspond to the areas where the tool pin directly contacts the material, whereas region (2) represents the area contacted by the lower part of the tool pin. Upon examining the cross-sectional micrograph of the Experiment 6 specimen (Figure 11d), the presence of cracks is observed. In a study by Cakan and Uğurlu on the FSSW joining of DP600 steel and AA7075-T6, it was reported that cracks that formed along the walls may occur during the solidification of partially melted regions [71].

### 3.4. Bending Test Results After FSSW

**Figure 12:**
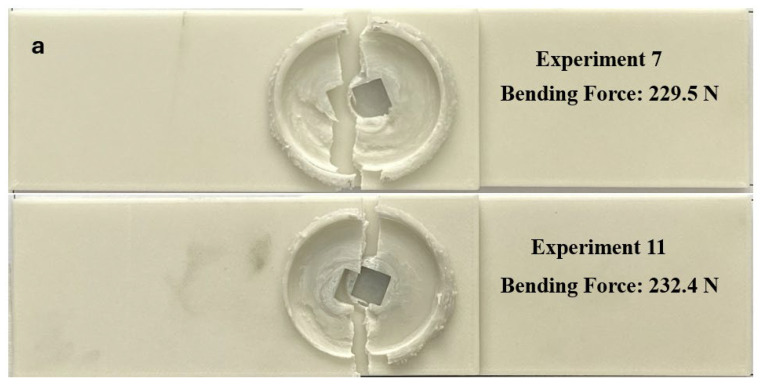

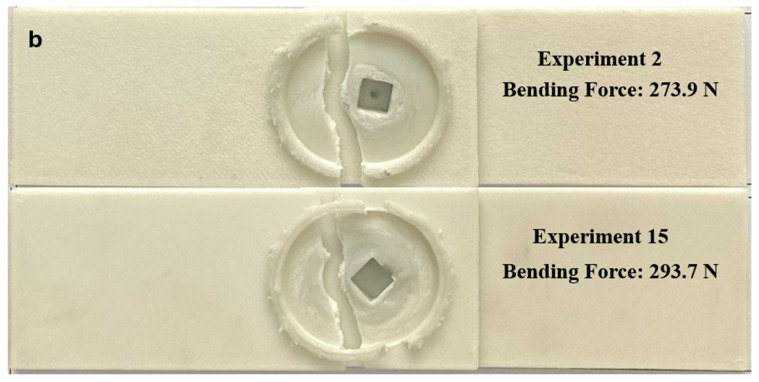
Fracture surfaces after bending test: (**a**) Experiment 7 (20 mm/min and 1200 rpm) and Experiment 11 (30 mm/min and 900 rpm); (**b**) Experiment 2 (10 mm/min and 1200 rpm) and Experiment 15 (30 mm/min and 2100 rpm).

Figure 12a presents the specimens with the lowest bending force (Experiment 7 and Experiment 11), while Figure 12b shows those with the highest bending force (Experiment 2 and Experiment 15). Fracture occurred in all plates as a result of bending. In Experiments 7 and 11, fracture initiated from the nugget region, whereas in Experiments 2 and 15, it occurred in the stir zone. In all specimens, the applied bending force led to significant deformation.

**Figure 13:**
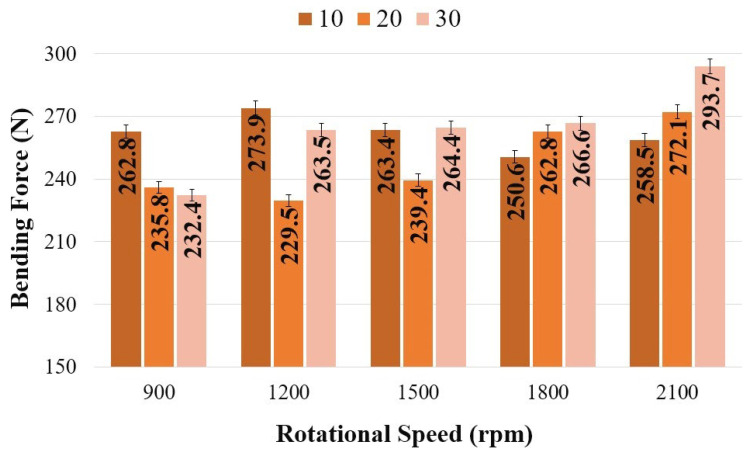
Bending force values as a function of tool rotational speed and plunge rate.

Figure 13 presents the variation in bending force as a function of tool rotational speed and plunge rate. The highest bending force was determined as 293.7 N at a tool rotational speed of 2100 rpm and a plunge rate of 30 mm/min. At a rotational speed of 900 rpm, the bending forces were measured as 262.8 N, 235.8 N, and 232.4 N for plunge rates of 10, 20, and 30 mm/min, respectively. At this speed, the highest value was obtained at 10 mm/min, while the lowest value (232.4 N) was recorded at 30 mm/min. At 1200 rpm, the bending force values were 273.9 N at 10 mm/min, 229.5 N at 20 mm/min, and 263.5 N at 30 mm/min. At 1500 rpm, the lowest bending force was recorded at 20 mm/min with 239.4 N, whereas at 30 mm/min, the value increased to 264.4 N. At 1800 rpm, the bending forces were obtained as 250.6 N, 262.8 N, and 266.6 N for 10, 20, and 30 mm/min, respectively, indicating an increase in bending force with increasing plunge rate. A similar trend was observed at 2100 rpm, where the bending forces increased with plunge rate, reaching 258.5 N at 10 mm/min, 272.1 N at 20 mm/min, and 293.7 N at 30 mm/min.

## 4. Results

With the increasing prevalence of 3D printing applications, various joining methods have been explored for assembling components produced by additive manufacturing. Among these methods, FSSW has emerged as one of the most suitable alternatives to conventional resistance spot welding and riveting, particularly for joining lightweight structural metals such as aluminum and magnesium alloys in the automotive and aerospace industries. While FSSW has been applied to the joining of various polymer materials, parameter optimization becomes particularly critical in fiber-reinforced systems, as the fiber distribution can be significantly affected during the process. Therefore, the selection of appropriate processing parameters is essential to ensure efficient and reliable FSSW performance when working with such materials. In this study, the weldability of glass fiber-reinforced PLA materials using FSSW was investigated. The effects of three different plunge rates and five different tool rotational speeds on the weld zone were examined.

The literature indicates that increasing tool rotational speed and dwell time enhances frictional interaction, thereby increasing heat generation and consequently affecting weld quality. In addition, the plunge rate also influences the friction between the tool and the material. Therefore, understanding the interaction between tool rotational speed and plunge rate, and selecting appropriate values, is of critical importance. Consistent with the literature, three distinct zones (stir zone, mixed zone, and shoulder zone) were observed after the FSSW process. At tool rotational speeds of 900 rpm, 1200 rpm, and 2100 rpm, the weld force varied depending on the changes in plunge rate. However, at 1500 rpm and 1800 rpm, it was determined that variations in plunge rate did not result in significant changes in weld force.

The highest weld force was determined to be 930.7 N at a plunge rate of 20 mm/min and a tool rotational speed of 900 rpm. In the bending test, the maximum bending force was recorded as 293.7 N at a plunge rate of 30 mm/min and a tool rotational speed of 2100 rpm.

Under unsuitable FSSW parameters, defects were observed at the lower part of the stir zone. In addition, excessive heat generation in the weld region led to material expulsion from the tool shoulder.

3D-printed reinforced composite components show significant potential for use in industrial applications where lightweight structures and high strength are required. However, it should be noted that the properties of such materials may vary depending on printing parameters and the composition of filaments obtained from different manufacturers. Therefore, further research is needed to comprehensively evaluate the welding performance of 3D-printed materials.

## Figures and Tables

**Table 1:** Mechanical properties of the filament used [35].

Brand	eSUN
Color	White
Density (g/cm^3^)	1.31
Tensile Strength (MPa)	59.27
Elongation at Break (%)	7.99
Flexural Strength (MPa)	85.01
Flexural Modulus (MPa)	4414.89
Impact Strength (kJ/m^2^)	10.16

**Table 2:** Printing parameters of the samples.

Infill Ratio (%)	100
Infill Pattern	Linear
Layer Thickness (mm)	0.2
Nozzle Diameter (mm)	0.4
Walls Number	3
Nozzle Temperature (°C)	220
Bed Temperature (°C)	55
Print Speed (mm/s)	80
Raster Angle (°)	[−45, +45]

**Table 3:** FSSW parameters and their effects on weld quality.

Welding Parameter	Effect on the Weld	References
Rotational speed	It determines the heat input and material mixing; too low a speed leads to weak bonding, while too high a speed may cause excessive heating and defects. It is generally one of the primary critical parameters affecting tensile strength.	[38,42,43]
Plunge depth	It controls the welding area and mechanical interlocking; insufficient depth leads to weak adhesion, while excessive depth causes thinning and defects.	[38,40,42]
Plunge rate	It affects heat generation and cycle time. Low plunge rate may lead to excessive plastic deformation due to increased heat generation.	[44,45]
Dwell time	It directly determines the heat input and material flow in the weld zone. A short dwell time may lead to voids and a weak interface, while a longer dwell time can result in higher strength but carries the risk of excessive softening.	[46,47]
Shoulder diameter	It alters the generated heat and the deformation zone.	[48,49,50]
Stirring pin diameter and geometry	It determines the stirring volume, material flow, and nugget shape; an appropriate geometry provides a more homogeneous structure and higher strength.	[51,52]

**Table 4:** Parameters and levels used in FSSW.

Material	Welding Parameter	Reference
ABS—ABS	Rotational speed (rpm): 1000, 2000, 3000 rpm	[13]
	Plunge depth (mm): 10.5, 11, 11.5	
	Dwell Time (s): 20, 30, 40	
Carbon fiber-reinforced polyamide 66—Carbon fiber-reinforced polyamide 66	Rotational speed (rpm): 1000–3000	[14]
	Plunge depth (mm): 1.8–2.2	
	Friction Time (s): 3.6–7.5	
	Holding pressure time (s): 0–20	
HDPE—HDPE	Rotational speed (rpm): 700, 900, 1100	[9]
	Dwell Time (s): 20, 40, 60	
	Plunge depth (mm): 5.7, 6.2, 6.7	
PP—PP	Rotational speed (rpm): 700, 900, 1100	[53]
	Dwell Time (s): 50, 100, 150	
	Plunge depth (mm): 5.7, 6.2 and 6.7	
	Shoulder concavity angle: 0–12	
	Tool geometry: straight, tapered, threaded, square, triangular, hexagonal	
Dissimilar Material (PLA Plus and PLA CF)	Rotational speed (rpm): 1200	[41]
	Dwell Time (s): 10	
	Plunge depth (mm): 4.7	
	Plunge rate (mm/min): 10	
	Amplitude (%): 0, 30, 50, 70, 90, 100	

**Table 5:** FSSW parameters.

Material	Pin Geometry	Plunge Rate (mm/min)	Rotational Speed (rpm)
PLA-GF	Square	10	900
		20	1200
		30	1500
			1800
			2100

**Table 6:** FSSW experiment results.

Experiment Number	Plunge Rate (mm/min)	Rotational Speed (rpm)	Force (N)	Elasticity Modulus (MPa)	Elongation (%)	Welding Efficiency %
Ref.			726.75 ± 9.2	620.6 ± 54.9	4.92 ± 0.3	
1	10	900	898.3 ± 58.4	1552.1 ± 174.2	1.87 ± 0.4	123.6
2	10	1200	731.4 ± 58.4	1967.4 ± 116.4	1.87 ± 0.4	100.6
3	10	1500	832.8 ± 50.2	1820.6 ± 142.5	1.99 ± 0.2	114.5
4	10	1800	835.5 ± 70.8	1885.0 ± 341.5	1.85 ± 0.3	114.9
5	10	2100	795.9 ± 60.0	1802.0 ± 157.0	1.56 ± 0.2	109.5
6	20	900	930.7 ± 115.0	1432.4 ± 250.7	1.80 ± 0.2	128.0
7	20	1200	914.3 ± 56.4	1482.2 ± 197.5	1.95 ± 0.4	125.8
8	20	1500	863.2 ± 80.9	1353.8 ± 142.5	1.51 ± 0.2	118.7
9	20	1800	864.6 ± 52.8	1311.8 ± 219.9	1.98 ± 0.1	118.9
10	20	2100	925.4 ± 45.0	1570.5 ± 406.9	1.89 ± 0.1	127.3
11	30	900	772.3 ± 25.2	1805.8 ± 72.7	1.50 ± 0.1	106.2
12	30	1200	907.1 ± 111.9	1850.8 ± 71.6	1.76 ± 0.1	124.8
13	30	1500	837.4 ± 59.0	1680.8 ± 81.5	1.71 ± 0.1	115.2
14	30	1800	822.5 ± 76.2	1740.1 ± 138.5	2.11 ± 0.7	113.1
15	30	2100	869.8 ± 58.4	1552.1 ± 174.2	1.87 ± 0.4	119.6

## Data Availability

The original contributions presented in this study are included in the article. Further inquiries can be directed to the corresponding author.
